# The crosstalk between the gut microbiota and tumor immunity: Implications for cancer progression and treatment outcomes

**DOI:** 10.3389/fimmu.2022.1096551

**Published:** 2023-01-16

**Authors:** Man Wang, Lei Zhang, Wenguang Chang, Yuan Zhang

**Affiliations:** Institute for Translational Medicine, The Affiliated Hospital of Qingdao University, College of Medicine, Qingdao University, Qingdao, China

**Keywords:** gut microbiota, cancer pathogenesis, microbiota-derived metabolites, dysbiosis, tumor immunity, treatment outcomes

## Abstract

The gastrointestinal tract is inhabited by trillions of commensal microorganisms that constitute the gut microbiota. As a main metabolic organ, the gut microbiota has co-evolved in a symbiotic relationship with its host, contributing to physiological homeostasis. Recent advances have provided mechanistic insights into the dual role of the gut microbiota in cancer pathogenesis. Particularly, compelling evidence indicates that the gut microbiota exerts regulatory effects on the host immune system to fight against cancer development. Some microbiota-derived metabolites have been suggested as potential activators of antitumor immunity. On the contrary, the disequilibrium of intestinal microbial communities, a condition termed dysbiosis, can induce cancer development. The altered gut microbiota reprograms the hostile tumor microenvironment (TME), thus allowing cancer cells to avoid immunosurvelliance. Furthermore, the gut microbiota has been associated with the effects and complications of cancer therapy given its prominent immunoregulatory properties. Therapeutic measures that aim to manipulate the interplay between the gut microbiota and tumor immunity may bring new breakthroughs in cancer treatment. Herein, we provide a comprehensive update on the evidence for the implication of the gut microbiota in immune-oncology and discuss the fundamental mechanisms underlying the influence of intestinal microbial communities on systemic cancer therapy, in order to provide important clues toward improving treatment outcomes in cancer patients.

## 1 Introduction

The mammalian immune system is made up of an intricate network of innate and adaptive cells and has emerged as a key player in cancer development and prevention (1). Uncontrolled immune activation can trigger chronic inflammation. Of note, the causal link between chronic inflammation and carcinogenesis has been well established (2, 3). Natural killer (NK) cells and CD8^+^ T cells are main immune effector cells that cooperate to eliminate cancer cells (4, 5). Importantly, cancer cells have evolved multiple strategies to evade immune attack. Cancer cells can reduce antigenicity to disguise as noncancerous cells, hence avoiding the detection by cytotoxic T cells (6, 7). They restrict the expression of major histocompatibility complex class I (MHC-I) and costimulatory molecules, rendering cancer cells able to escape from T cell-mediated immunity (8). Cancer cells are capable of producing immunosuppressive factors and activating myeloid-derived suppressor cells (MDSCs), regulatory T (Treg) cells, and tumor-associated macrophages (TAMs) (9). These strategies contribute to the construction of an immune-tolerant tumor microenvironment (TME) that represses cytotoxic immune cell activity and facilitates cancer progression. The complex intercourse between cancer cells and the host immune system has a huge impact on cancer development. In recent years, tumor immunity has become a research hotspot in the field of oncology.

The response to cancer therapy dramatically varies among cancer patients (10–12). Intrinsic reasons behind heterogeneous clinical responses have not yet been illuminated. It is believed that host immune status and diverse environment factors have been closely interrelated with the clinical outcome in cancer patients (13, 14). Among these factors, the commensal microbiota has garnered increasing attention over the past decade. The gut microbiota comprises trillions of commensal microorganisms, which co-evolve with the host and carry out multitudinous functions including immune modulation, metabolism, natural defense against infection, and nutrient acquisition (15). Importantly, there is expanding evidence verifying the regulatory role of the gut microbiota in tumor immunity (16, 17). Concretely, beneficial commensal bacteria can strengthen innate and/or adaptive immunity, thus contributing to tumor inhibition. However, the altered gut microbiota in cancer reshapes the TME to drive cancer progression (18, 19). Furthermore, the gut microbiota can also modulate treatment response in cancer patients through its interaction with diverse immune cells (20). Therefore, modulation of the gut microbiota is potentially a tractable option to enhance antitumor immunity and amplify the anticancer potency of conventional treatment modalities. In this review, we summarize current knowledge linking the gut microbiota to the functionality of antitumor immune system and specifically highlight mechanistic explanations of the complex crosstalk between the gut microbiota and tumor immunity in cancer. Moreover, we review the influence of the gut microbiota on the efficacy of cancer therapies and discuss the potential clinical implication of the gut microbiota interventions in cancer treatment.

## 2 The gut microbiota

### 2.1 The composition and main functionalities of the gut microbiota

**Figure 1:**
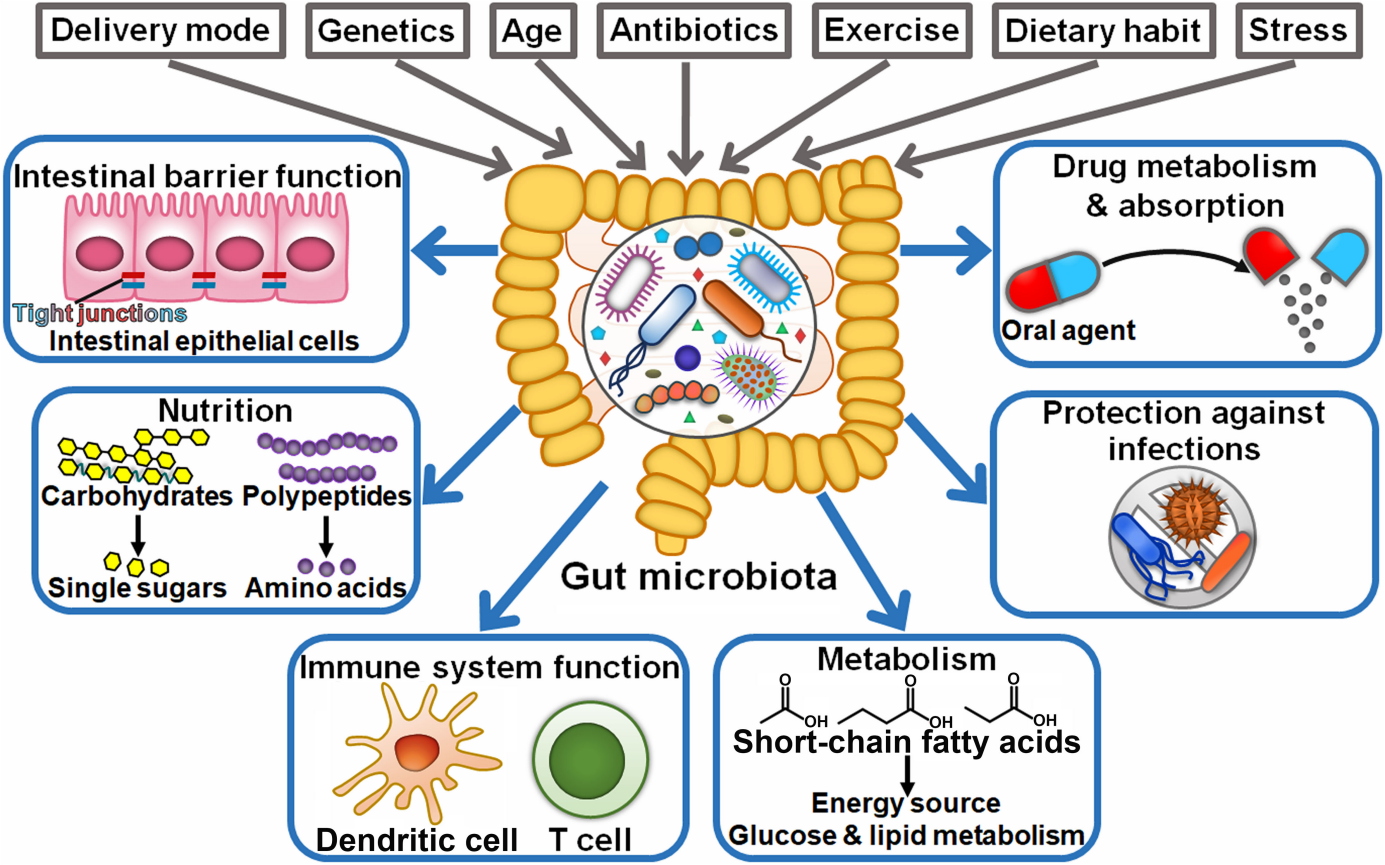
Schematic representation of the beneficial functions of the gut microbiota. Intrinsic and extrinsic factors can alter the composition of the gut microbiota, including the mode of delivery at birth, host genetics, age, antibiotics, exercise, dietary habit, and stress. The gut microbiota plays a critical role in human health, such as preservation of intestinal barrier integrity, breaking down food compounds (e.g., carbohydrates and proteins), supplying essential nutrients, and regulation of immune system development. Commensal microbes also coordinate energy, glucose and lipid metabolism. Importantly, they confer protection against invading pathogens. In addition, intestinal microbes have the ability to influence the bioavailability, absorption and therapeutic efficacy of oral agents.

The gut microbiota encompasses a large population of microorganisms (10-100 trillion) that dwell in the human gastrointestinal tract (21). The gut microbiota is established at birth and remains for the whole life, while it commonly undergoes dynamic variations (22). The factors influencing the composition and diversity of the gut microbiota include delivery mode, host genetics, age, antibiotic intake, dietary habit, exercise, and stress (23) (**Figure 1**). This dynamic community mainly comprises species from the prokaryotic domain (archaea and bacteria), fungi, protists, and viruses (24). Bacteroidetes (e.g., *Bacteroides*) and Firmicutes (e.g., *Clostridium*, *Enterococcus*, and *Lactobacillus*) are the most common (90% of the total) phyla in the gut microbiota (25). Other phyla, such as Actinobacteria, Fusobacteria, Proteobacteria, and Verrucomicrobia, are consistently present in the human gut (26). The gut microbiota can interact with host cells to regulate various physiological processes including preservation of intestinal barrier integrity, nutritional acquisition, immune system function, metabolism and host defense against infections (27). Moreover, intestinal microbes can influence the bioavailability and absorption of oral agents and result in the formation of pharmacologically active metabolites that further aggrandize the effectiveness or side effects of these agents (28). The compositional and functional change in the gut microbiota, known as dysbiosis, is involved in the pathogenesis of various diseases, especially cancer (29–31).

### 2.2 The roles of the gut microbiota in host immune development

There is mounting evidence that the gut microbiota plays a key role in the development of both the intestinal and systematic immune system (32). The host immune system in turn remodels the structure and function of the gut microbiota (33). The effects of commensal microbiota on the host immune system are strongly established by using germ-free (GF) animal models. Early studies indicated that absence of commensal microbes contributed to defective lymphoid tissue architecture, reduced numbers of intestinal lymphocytes and decreased production of antimicrobial peptides and immunoglobulin A (IgA) (27). GF mice exhibited a marked decrease in the number of αβ and γδ intra-epithelial lymphocytes (IELs) compared to normal mice, which could be restored by the introduction of microbes (34). Invariant natural killer T (iNKT) cells from GF mice showed an immature phenotype and impaired activation following antigen exposure (35). Antigen-stimulated CD8^+^ T cells were incapable of transiting into a memory phenotype in GF mice (36). Commensal microbiota-derived short-chain fatty acid (SCFA) strengthened memory potential of activated CD8^+^ T cells. Segmented filamentous bacteria might be required for the differentiation of CD4^+^ T helper 17 (Th17) effector cells in the mucosa of the small intestine (37). Mazmanian et al. (38) revealed that the polysaccharide from the commensal *Bacteroides fragilis* drove the cellular and physical maturation of the developing immune system in mice. Extracellular signals from commensal microbes that regulated intestinal Ig repertoires could direct B cell development in the intestinal mucosa (39). Toll-like receptor 5 (TLR5) expression in the gut epithelium of neonatal mice shaped the composition of the gut microbiota through counter-selection of colonizing flagellated bacteria, which in turn affected immune homeostasis and health in adulthood (40).

On the contrary, host immunity performs regulatory effects on intestinal microbial community. Mice deficient in the transcription factor T-bet had an intestinal microbial population related to a colitogenic phenotype (41). Likewise, loss of the bacterial flagellin receptor TLR5 altered the gut microbiota composition in mice (42). Mice deficient for colonic epithelial cell expression of nucleotide-binding oligomerization domain-like receptor (NLR) family pyrin domain containing 6 (NLRP6) were characterized by the overgrowth of the bacterial phyla Bacteroidetes and Saccharibacteria (43). Knockdown of myeloid differentiation factor 88 (MyD88) induced an expansion of segmented filamentous bacteria and a reduced Firmicutes/Bacteroidetes ratio in mice (44, 45). Depletion of interleukin-22 (IL-22)-producing innate lymphoid cells (ILCs) selectively orchestrated colonization of *Alcaligenes* species to restrict systemic inflammation, and this effect was attenuated by IL-22 administration (46). Recombination-activating genes (Rag1 and Rag2) play a prominent role in adaptive immunity (47). Compared with wide-type (WT) mice, mice deficient in Rag1 and Rag2 exhibited a greater proportion of *Akkermansia muciniphila* and segmented filamentous bacteria, respectively (48, 49). Altogether, adaptive immunity has a critical role in shaping the composition and diversity of the gut microbiota. It is worth pointing out that most of the aforementioned results are generated based on gene knockdown animals that lack specific immune-related genes. Defects in the immune system may alter colonic microbiota that in turn coordinates host immune responses to restore or exacerbate the defects. Therefore, it is necessary to understand the impact of specific immune cells or factors on the gut microbiota composition in animal models with sound and mature immune systems.

Collectively, it is universally accepted that the reciprocal interaction between intestinal microbiota and the host immune system have profound impacts on immune development and function. The mechanisms through which the gut microbiota modulates host immune responses may involve regulation of microbial translocation, delivery of their components and their metabolites into the circulation, and activation of epithelial, immune, or stromal cells within the gut. At present, intestinal microbiota–immunity interactions are far from fully defined and warrant in-depth investigations. A sophisticated understanding of this extensive bidirectional communication may be instrumental in elucidating the mechanisms underlying the development of the host immune system and provide new insights into more effective therapeutic approaches for the treatment of immune-related disorders.

## 3 The gut microbiota and innate immunity

### 3.1 Antitumor innate immunity

#### 3.1.1 Innate immune cells

The gut microbiota can affect cancer pathogenesis through regulation of innate immune cells (**Table 1**). Disruption of the gut microbiota *via* an antibiotic cocktail (ABX) consisting of vancomycin, neomycin, metronidazole, amphotericin, and ampicillin (VNMAA) accelerated breast cancer growth (50). VNMAA treatment had no effects on tumor growth in GF mice, intimating the significance of the gut microbiota in controlling cancer development. VNMAA treatment apparently reduced the abundance of *Faecalibculum rodentium* that exhibited an anti-tumorigenic potential (77). As expected, *F. rodentium* supplementation delayed tumor growth in VNMAA-treated mice, alluding to the protective role of *F. rodentium* in breast cancer progression. The abundance of mast cells was increased in the tumor stromal regions of VNMAA-treated mice. VNMAA-induced stromal mast cells impelled breast cancer development. Due to the association between the gut microbiota and mast cell function (78), it is necessary to delve into the exact effects of antibiotic-induced depletion of beneficial gut microbes on mast cell homing and functionality. Future studies should focus on understanding how the gut microbiota contributes to tumor growth control *via* regulation of mast cells.

**Table 1:** The regulatory effects of gut microbiota and its metabolites on tumor immunity.

Cancer type	Gut microbiota/microbial metabolites	Effects	Mechanisms	References
Breast cancer	Commensal microbiota	Induction of antitumor innate immunity	Reduce the abundance of stromal mast cells	(50)
Melanoma	Commensal microbiota	Induction of antitumor innate immunity	Induce the expansion of NK cells and Th1 cells and foster their homing to the bone marrow	(51)
Human papilloma virus-induced cancer	*Lactobacillus casei* BL23	Induction of antitumor innate immunity	Enhance the proliferation and cytotoxic activities of NK cells	(52)
Lymphoma	C-di-AMP, *Akkermansia muciniphila*	Induction of antitumor innate immunity	Facilitate the activation and intratumoral infiltration of NK cells	(53)
Colorectal cancer	Bacteroides, Prevotella	Induction of antitumor innate immunity	Inhibit the production of IL-9 and IL-17A	(54)
Colorectal cancer	Commensal microbiota	Induction of antitumor innate immunity	Inhibit the production of IL-1β, IL-6 and TNF-α; promote the production of IL-10 and TGF-β; foster Treg cell accumulation	(55)
Colorectal cancer	*Odoribacter splanchnicus*	Induction of antitumor innate immunity	Induce the expansion of Th17 cells and increase the levels of IL-17A and IL-22	(56)
Colorectal cancer	Sodium butyrate	Induction of antitumor innate immunity	Reduce the frequency of Treg cells and increase the frequency of NKT and Th17 cells; promote IL-17 production and suppress IL-10 production	(57)
Hepatocellular carcinoma	*Lactobacillus reuteri*, acetate	Induction of antitumor innate immunity	Inhibit IL-17A expression by ILC3s	(58)
Colorectal cancer	Malnutrition-induced gut microbiota dysbiosis	Activation of pro-tumor innate immunity	Facilitate the activation of macrophages	(59)
Colorectal cancer	*Proteus mirabilis*	Activation of pro-tumor innate immunity	Inhibit hepatic accumulation of Kupffer cells	(60)
Cholangiocarcinoma	Gram-negative commensal bacteria	Activation of pro-tumor innate immunity	Promote hepatic accumulation of PMN-MDSCs	(61)
Hepatocellular carcinoma	Vancomycin-induced gut microbiota dysbiosis	Activation of pro-tumor innate immunity	Activate M2 macrophages *via* IL-25	(62)
Pancreatic ductal adenocarcinoma	Commensal microbiota from *Rag1*^-/-^ mice	Activation of pro-tumor innate immunity	Inhibit the cytotoxicity and migration of NK cells	(63)
Colorectal cancer	*Peptostreptococcus anaerobius*	Activation of pro-tumor innate immunity	Increase the levels of IFN-γ and IL-10; promote the intratumoral infiltration of MDSCs, granulocytic tumor-associated neutrophils and TAMs	(64)
Colorectal cancer	*Fusobacterium nucleatum*	Activation of pro-tumor innate immunity	Increased the levels of CXCL1, Eotaxin, IFN-γ, IL-6, IL-9, IL-12, IL-17A, MCP-1 and TNF-α; promote the accumulation of Treg cells and MDSCs; suppress the intratumoral infiltration of Th17 cells, NK cells, CD4^+^ T cells and CD8^+^ T cells	(65)
Colon cancer	*Candida albicans*	Activation of pro-tumor innate immunity	Enhance IL-7 production by macrophages and increase IL-22 level	(66)
Colorectal cancer	*Helicobacter hepaticus*	Induction of T cell immunity	Activate CD4^+^ Tfh cells and facilitate the maturation of peritumoral tertiary lymphoid structures	(67)
Colorectal cancer	*Akkermansia muciniphila*	Induction of T cell immunity	Facilitate the expansion and activation of CTLs	(68)
Colon cancer	*Alistipes senegalensis*, *Bacteroides dorei*, *Bacteroides uniformis* JCM 5828, *Eubacterium limosum*, *Fusobacterium ulcerans*, *Parabacteroides distasonis*, *Parabacteroides gordonii*, *Parabacteroides johnsonii*, *Paraprevotella xylaniphila*, *Phascolarctobacterium faecium* and *Ruminococcaceae bacterium* cv2	Induction of T cell immunity	Increase the frequency of IFN-γ^+^ CD8 TILs	(69)
Colon adenocarcinoma	Commensal microbiota from B cell-defective mice	Induction of T cell immunity	Foster the expansion of CD8^+^ T cells	(70)
Colorectal cancer, melanoma	*Anaerostipes caccae*, *Eubacterium hallii*, *Faecalibacterium prausnitzii* and *Roseburia intestinalis*	Induction of T cell immunity	Enhance the intratumoral infiltration and activation of CD8^+^ T cells	(71)
Melanoma	Commensal microbiota from *Rnf5^-/-^* mice	Induction of T cell immunity	Increase the frequency of TILs and improve antitumor cytokine response	(72)
Breast cancer, hepatocellular carcinoma	*Escherichia coli* strain Nissle 1917	Induction of T cell immunity	Promote tumor-specific effector T cell infiltration and induce DC maturation	(73)
Cutaneous melanoma	*Acinetobacter, Flammeovirga, Gelidibacter and Lachnoclostridium*	Induction of T cell immunity	Increase the levels of tumor-infiltrating CD8^+^ T cells and chemokines	(74)
Colorectal cancer	Gut microbiota dysbiosis	Suppression of T cell immunity	Induce T cell exhaustion	(75)
Melanoma, lung carcinoma	Antibiotic-induced gut microbiota dysbiosis	Suppression of T cell immunity	Reduce the count of intratumoral effector T cells and decrease the serum level of TNF-α	(76)
Hepatocellular carcinoma	Commensal microbiota	Suppression of T cell immunity	Induce the expansion of IL-10^+^ Treg cells and inhibit the expansion of cytotoxic CD8^+^ T cells	(16)

NK cells are key ILCs mediating tumor immunosurveillance and elimination (79). The gut microbiota was reported to blunt melanoma bone growth (51). Gut microbiota depletion by broad-spectrum antibiotics (ATB) promoted intraosseous melanoma growth by blocking melanoma-induced expansion of intestinal NK cells and Th1 cells and their egress from the gut into the bone marrow of tumor-bearing bones. Suppression of sphingosine-1-phosphate (S1P)-mediated migration of NK cells and Th1 cells, or blockade of C-X-C chemokine receptor 3 (CXCR3)/C-X-C motif chemokine ligand 9 (CXCL9)-mediated trafficking to the bone marrow, alleviated the expansion of these immune cells and thus promoted melanoma growth. These observations unveiled the microbiota-dependent gut-bone crosstalk in melanoma-bearing mice. As microbiota depletion impels melanoma progression, special attention should be addressed to the adverse outcomes of antibiotic-induced intestinal dysbiosis. *Lactobacillus casei* BL23 displayed an antagonistic effect against human papilloma virus (HPV)-induced cancer (52). In terms of mechanism, *L. casei* BL23 induced a significant increase in the level of anticarcinogenic IL-2. The level of IL-2 was negatively associated with tumor size in HPV-induced cancer model. Blockade of IL-2 generation using neutralizing monoclonal antibodies resulted in a decreased level of T cells and opposed the protective effect of *L. casei* BL23. IL-2 can facilitate the proliferation and cytotoxic activities of NK cells (80). Consistently, *L. casei* BL23 had the ability to recruit NK cells with high cytotoxic activities toward cancer cells. Furthermore, preventive administration of *L. casei* BL23 suppressed cancer occurrence *in vivo* through systemic recruitment of NK cells, while therapeutic administration remarkably inhibited cancer cell growth through upregulation of IL-2 and activation of T cells. Altogether, *L. casei* BL23 may represent a promising agent for cancer prevention and treatment. Lam et al. (53) revealed that gut microbiota-derived signals, especially stimulator of interferon genes (STING) agonist cyclic di-adenosine monophosphate (c-di-AMP), induced type I interferon (IFN-I) production in intratumoral monocytes, which contributed to the attraction and activation of NK cells. Active NK cells then produced C-C motif chemokine ligand 5 (CCL5) and X-C motif chemokine ligand 1 (XCL1) to recruit DCs, which in turn stimulated NK cells *via* IL15/IL15 receptor (IL-15R), leading to a positive feedback loop to potentiate antitumor immunity. IFN-I receptor deficiency or antibiotic treatment impaired the response to oxaliplatin therapy in lymphoma-bearing mice. Importantly, absence of microbiota did not cause a marked alternation in tumor burden, suggesting that microbiota-derived signals could not prevent tumor growth but instead orchestrated innate immunity for preferable response to cancer treatment (53). Monocolonization of *A. muciniphila* was sufficient to boost antitumor immune response and increased susceptibility of GF mice to oxaliplatin exposure by activating the STING/INF-I/NK cell/DC axis. Consistently, microbiota affected the responses of patients with advanced melanoma to immune checkpoint blockade (ICB) by modifying the innate immune TME. The levels of monocytes, IFN-I, DCs, and IL-15 receptor subunit α (IL-15RA) were positively associated with better clinical outcomes in patients with advanced melanoma post ICB (53). GF mice receiving microbiota from ICB responders displayed skewing of monocytes toward inflammatory (antitumorigenic) macrophages. Oppositely, mononuclear phagocytes in the TME of mice receiving microbiota from ICB nonresponders differentiated into suppressive (tumor-promoting) macrophages. Mice that received ICB nonresponder-derived fecal microbiota transplantation (FMT) presented decreased proportion of intratumoral DCs, monocytes, and NK cells, as well as reduced levels of IFN-β1 in tumor tissues. As expected, microbiota-mediated reprograming of the TME led to increased tumor burden in nonresponder FMT mice. Furthermore, clinical trials with a similar FMT-ICB design provided a solid cause-effect relationship between microbiota, especially *A. muciniphila*, the IFN-I signaling and sensibility to ICB. IFN-I-producing monocytes are important modulators of innate immune system, stressing their potential as therapeutic targets to reinforce antitumor immunity. The specific microbes that regulate IFN-I production in monocytes should be defined. It is intriguing where and how mononuclear phagocytes sense microbiota-derived signals. The mechanism through which microbial STING agonist specifically reprograms TME merits further study.

#### 3.1.2 Inflammatory response

Intestinal microbes have emerged as an important modulator of inflammatory response. Tumor tissues from colorectal cancer (CRC) patients presented higher percentages of Th2, Th17 and Treg cells than healthy mucosa samples (54). Moreover, the levels of interferon-γ (IFN-γ), IL-1α, IL-1β, IL-2, IL-6, IL-8, IL-9, IL-10, IL-17A, macrophage chemoattractant protein-1 (MCP-1), macrophage inflammatory protein-1α (MIP-1α), P-selectin and tumor necrosis factor-α (TNF-α) were significantly increased in CRC samples in comparison with healthy mucosa samples. CRC samples were enriched in Fusobacteria, Proteobacteria, and *Ruminococcus*. Moreover, the relative level of *Prevotella* spp. showed an inverse association with IL-17A and positively correlated with IL-9 in CRC samples. The abundance of *Bacteroides* spp. was negatively connected with IL-9. These results hinted the interplay between the commensal gut microbiota and the host immune system. IL-9 and IL-17A may exert both pro- and antitumor effects (81–83). It is necessary to corroborate whether these two cytokines are implicated in gut dysbiosis-induced colorectal carcinogenesis. The roles of *Bacteroides* spp. and *Prevotella* spp. in immune regulation and CRC progression are still poorly understood. The previous study revealed that *B*. *fragilis* restrained the development of IL-9-producing Th17 cells (84), while Prevotellaceae could favor Th17 cell differentiation (85). Despite these encouraging results, further study will be required to clarify how the gut microbiota modulates the cytokine signature in CRC patients. Another study showed that the level of Firmicutes was decreased while that of Bacteroidetes was increased in mice with colitis-associated CRC (CAC) (55). The amounts of these two phyla were restored following FMT treatment. Moreover, FMT treatment exhibited superiority in rehabilitating intestinal microbiota diversity. Remarkably, FMT suppressed the production of proinflammatory cytokines IL-1β, IL-6, and TNF-α in the colon tissues of CAC mice while facilitated the production of anti-inflammatory cytokines IL-10, and transforming growth factor-β (TGF-β), in part, by impeding canonical nuclear factor-κB (NF-κB) activity and colonic cell proliferation. In addition, FMT treatment triggered the accumulation of Treg cells, thus blunting inflammatory responses in CAC mice. These observations provided a potential therapeutic strategy for the intervention of intestinal cancer. Nevertheless, the dynamic alternation of the gut microbiota during CAC development is an important future question to pursue. The efficacy and safety of FMT treatment still merit further investigation. Transforming growth factor-β-activated kinase 1 (TAK1) is a key component of both innate and adaptive immune signaling cascades (86). Specific knockout of TAK1 in myeloid cells rendered mice resistant to DSS-induced CRC (56). Myeloid-specific TAK1-deficient mice had an enrichment of *Anaerophaga thermohalophila*, *Bacteroides uniformis*, *Eubacterium ventriosum* and *Odoribacter splanchnicus* and a reduction of *A. muciniphila*, *Bifidobacterium longum*, *Lactobacillus mucosae*, and *Streptococcus anginosus*. The altered gut microbiota might be linked to CRC development. Importantly, monocolonization of *O. splanchnicus* induced the expansion of intestinal Th17 cells and increased the amounts of IL-17A and IL-22 in the colon and serum of GF mice and ABX-treated mice. As expected, *O. splanchnicus* inoculation inhibited the growth of colon cancer in GF mice and ABX-treated mice. Thus, *O. splanchnicus* instigated protective immune response against CRC by activating Th17 cells and promoting innate immune cytokine production.

Gut microbiota-derived metabolites can affect cancer development, which can be attributed to their prominent immunoregulatory function. Reportedly, sodium butyrate (NaB), a main product of gut microbial fermentation, was able to prevent CRC liver metastasis (CLM) in mice (57). NaB supplementation reversed gut microbiota dysbiosis by elevating the proportions of Bacteroidetes and Firmicutes and reducing the ratio of Firmicutes to Bacteroidetes. NaB treatment reduced the abundance of harmful bacteria, including *Alistipes*, *Bacteroides*, Clostridiales, Desulfovibrionaceae and *Helicobacter* in CLM mice. In addition, NaB increased the abundance of SCFA-producing bacteria, such as *Eubacterium xylanophilum* and *Roseburia*. NaB treatment resulted in a decline in the frequency of Treg cells and an increase in the frequency of NK T (NKT) cells and Th17 cells in CLM mice liver. NaB increased the level of IL-17 while decreased that of IL-10 in CLM mice liver. As a result, NaB prevented tumor progression by modulating the gut microbiota and triggering host immune responses in CLM mice. It should be noted that the utility of NaB as an adjuvant to CLM therapy awaits clinical evidence. Because of the gut-liver axis, intestinal toxins and microbes can reach the liver *via* the portal vein, which allows the metabolism of gut-derived microbial products and nutrients in the liver. It is not surprising that the gut microbiota regulates the development of liver cancer. The relative abundance of *Lactobacillus reuteri* was decreased in hepatocellular carcinoma (HCC)-bearing mice compared with control mice (58). HCC mice displayed a substantial reduction in the serum concentration of microbial metabolites including acetate and valerate. Oral gavage with *L. reuteri* or the gut microbiota from control mice restored the serum level of acetate and retarded tumor growth in HCC recipient mice. Oppositely, antibiotic treatment antagonized the anticancer effect of *L. reuteri* in HCC mice. IL-17A was reported to induce tumor angiogenesis (87, 88). Consistently, HCC mice displayed a robust increase in IL-17A expression in hepatic type 3 innate lymphoid cells (ILC3s) (58). Moreover, tumor-infiltrating ILC3s were a poor prognosis factor in HCC patients. Transplantation of *L. reuteri* or acetate administration decreased IL-17A expression by ILC3s in HCC mice. Mechanistically, acetate suppressed ILC3 function by blocking histone deacetylase (HDAC) activity, promoting the acetylation of sex-determining region Y (SRY)-box 13 (Sox13) at site K30 and reducing Sox13 expression. In addition, acetate synergized with programmed death-1 (PD-1)/programmed death-ligand 1 (PD-L1) blockade and exerted strong anticancer effects against HCC *in vivo*. Regulating gut microbiota-derived metabolites may be a promising strategy to reinforce immunotherapy efficacy. The direct association between *L. reuteri* and IL-17A-producing ILC3s in HCC patients should be determined. *L. reuteri* may generate other bioactive metabolites that have the capacity to modulate antitumor immunity and tumor progression, which needs more attention. IL-17 plays dichotomous roles in tumor development. IL-17 increases the expression of CXCL1, CXCL5, CXCL6, CXCL8, and IL-6, which potentiate the immunosuppressive function of MDSCs, inhibit the intratumoral infiltration of T cells and recruit tumor-promoting macrophages and neutrophils (89). IL-17 has tumor-promoting effects by shaping immunosuppressive TME. Paradoxically, IL-17 can enhance antitumor immunity by recruiting effector cells (e.g., CD8^+^ T cells) into the TME (90). Host factors that determine the beneficial or detrimental role of IL-17 in cancer should be identified. Certain bacteria-derived signals increase IL-17 level while some microbial products exert an opposite role. It is of great significance to explore the overall effect of the gut microbiota on IL-17 production.

The inflammatory response has been considered as a driver of cancer progression. Inflammation may cause alternations in the gut microbiota and foster microbial migration to tumor tissues, which enhances the expression of proinflammatory cytokines and contributes to tumor deterioration (91). Proinflammatory cytokine production by immune cells constitutes a critical pro-tumorigenic mechanism that provides cancerous cells with a constant supply of growth and survival signals (92). Cytokines released by tumor-infiltrating immune cells motivate key transcription factors NF-κB and signal transducer and activator of transcription 3 (STAT3) that impel cancer progression *via* chemokine induction. Intestinal microbes and their components can antagonize proinflammatory cytokine response. Mechanistically, the gut microbiota controls host cytokine pathways and activates microbial metabolite sensors that are abundantly present on inflammatory cells (93). The possibility of targeting inflammation-related gut microbiota with some measures, including FMT, prebiotics, probiotics and symbiotics, to treat cancer is quiet attractive. The crosstalk between the gut microbiota and host cytokine pathways is highly complex and dynamic, thus calling for further studies to identify the anti-inflammatory components of the gut microbiota in cancer. The gut microbiota-cytokine interaction patterns may be cytokine-specific, cancer type-specific and microbe-specific. Additional efforts are critically needed to uncover the mechanisms that underlie gut microbiota-mediated regulation of inflammatory responses.

### 3.2 Pro-tumor innate immunity

#### 3.2.1 Innate immune cells

Chao et al. (59) discovered a significant alternation in intestinal microbiota (e.g., *Atopobium vaginae*, *Faecalibacterium prausnitzii*, and *Selenomonas sputigena*) among CRC patients with distinct nutritional conditions. Particularly, the high level of *A*. *vaginae* in tumor tissues was correlated with poor prognosis in CRC patients. Transplantation of fecal microbiota from malnourished CRC patients induced intestinal mucosal immunity in mice through attraction of B cells to stimulate macrophages. Importantly, the gut microbiota from malnourished CRC patients drove CRC progression in the dextran sodium sulfate (DSS)/azoxymethane (AOM) mouse model. Depletion of B cells markedly inhibited M2b macrophage polarization and attenuated the protumorigenic capacity of TAMs, consequently preventing CRC development triggered by malnutrition-induced gut microbiota dysbiosis. Taken together, the gut microbiota in CRC patients under malnutrition conditions was capable of promoting CRC development by affecting the activity of B cells and macrophages. The microbial species that regulate the activation of immune cells should be characterized and validated. It is intriguing how B cells activate macrophage *via* the gut microbiota in CRC with malnutrition. *In vivo* animal studies are necessary to verify the tumor-promoting role of *A*. *vaginae*. Supplementation of *Proteus mirabilis* could promote CLM in mice by diminishing the proportion of Kupffer cells in the liver (60). *In vitro* experiments further proved that *P. mirabilis* promoted CRC cell migration by repressing Kupffer cell proliferation. Collectively, *P. mirabilis* had the ability to reshape the liver immune microenvironment. The Kupffer cell subtype affected by *P. mirabilis* should be identified. It is not clear whether *P. mirabilis* affects other cells of hepatic immune response. The exact mechanism responsible for the immunoregulatory effect of this bacterial species awaits additional studies.

Neomycin and vancomycin showed significant antibacterial activity against Gram-negative and Gram-positive bacteria, respectively (61). Elimination of Gram-negative bacteria by neomycin inhibited the hepatic accumulation of CXCR2^+^ polymorphonuclear MDSCs (PMN-MDSCs) in murine cholangiocarcinoma models. Consequently, depletion of Gram-negative bacteria remarkably restrained cholangiocarcinoma growth. Conversely, transplantation of Gram-negative bacteria into GF mice promoted cholangiocarcinoma growth. Lipopolysaccharide (LPS), a main component of Gram-negative bacteria, was sufficient to recruit PMN-MDSCs to the liver. As expected, specific knockout of TLR4 in hepatocytes dramatically decreased PMN-MDSCs and retarded cholangiocarcinoma growth. Deficiency of CXCL1, a major ligand of CXCR2, also inhibited hepatic CXCR2^+^ PMN-MDSC accumulation. Thus, LPS/TLR4/CXCL1 mediated the effect of Gram-negative bacteria on PMN-MDSCs. The involvement of other TLRs in hepatic PMN-MDSC recruitment by Gram-negative bacteria needs to be determined in future studies. Altogether, Gram-negative gut bacteria directed hepatocytes to constitute an immunosuppressive microenvironment to accelerate cholangiocarcinoma progression. In addition, increased hepatic MDSCs also facilitated HCC growth (61). Continual research efforts are required to clarify whether hepatic MDSCs have a pro-tumor role in all liver cancer types.

The antibiotic vancomycin promoted the enrichment of Gram-negative bacteria (e.g., Deferribacteres, Proteobacteria, and Verrucomicrobia) and reduced the population of Gram-positive bacteria (e.g., Firmicutes) (62). Vancomycin-induced intestinal dysbiosis caused an increase in the levels of IL-25 in the serum and tissues of HCC patients, which activated M2 macrophages in TME. IL-25-induced M2 macrophages facilitated the growth, migration, and invasion of HCC cells *via* release of CXCL10 and induction of the epithelial-mesenchymal transition (EMT) process. In addition, upregulation of IL-25 was associated with poor prognosis in HCC patients. It was likely that antibiotic-induced dysbiosis of the gut microbiota triggered hyperplasia of intestinal epithelial tuft cells, concomitant with increased IL-25 secretion. Collectively, IL-25 may be a prospective therapeutic target for HCC management. It is intriguing how the gut microbiota coordinates the cytokine level and immune cell function. The exact effect of gut microbiota dysbiosis on antitumor immunity in HCC patients requires additional study.

The presence of the gut microbiota was associated with reduced intratumoral infiltration of NK cells in *Rag1*^-/-^ mice with pancreatic ductal adenocarcinoma (PDAC) (63). The gut microbiota also inhibited the activation of intratumoral NK cells, as evidenced by decreased production of IFN-γ. As expected, transplantation of the gut microbiota derived from *Rag1*^-/-^ mice into GF mice accelerated PDAC growth. Depletion of NK cells abrogated the antitumor effect of gut microbiota ablation in both immunocompetent and immunodeficient mouse models, alluding to the role of NK cells in gut microbiota-mediated PDAC development. The absence of microbiota in PDAC tissues suggested that the pro-tumor effect of the gut microbiota was independent of the direct interaction between the gut microbiota and TME. Consistently, the abiotic culture supernatant from *Rag1*^-/-^ mice feces could restrain the cytotoxicity and migration of NK cells. Collectively, gut microbiota-derived components promoted PDAC progression by regulating the activity of intratumoral NK cells. Additional work is required to dissect specific microbial species and their components that could modulate PDAC progression through interaction with tumor-infiltrating NK cells. Manipulation of the gut microbiota to regulate innate immunity may represent a new approach for PDAC intervention.

#### 3.2.2 Inflammatory response

*Peptostreptococcus anaerobius*, an anaerobic bacterium enriched in intestinal microbiota from CRC patients, was reported to promote CRC development in *Apc*^Min/+^ mice (64). Mechanistically, the surface protein of *P. anaerobius*, putative cell wall binding repeat 2 (PCWBR2), could directly combine with CRC cells *via* its receptor α2/β1 integrin, which instigated the phosphatidylinositol 3-kinase (PI3K)/protein kinase B (Akt) pathway *via* phosphor-focal adhesion kinase. These events led to enhanced cell proliferation and activation of NF-κB, which induced a proinflammatory response as manifested by elevated levels of IFN-γ and IL-10 in CRC tissues of *P. anaerobius*-treated *Apc*^Min/+^ mice. Moreover, the infiltrating levels of MDSCs, granulocytic tumor-associated neutrophils and TAMs were increased in *P. anaerobius*-treated *Apc*^Min/+^ mice. These infiltrating immune cells were linked with chronic inflammation and CRC development. Blockade of integrin α2/β1 repressed its interaction with *P. anaerobius* and abolished *P. anaerobius*-induced tumorigenic response. Therefore, the PCWBR2/integrin α2/β1 axis represented a promising therapeutic target for CRC intervention.

Administration of *Fusobacterium nucleatum* aggravated tumor liver metastasis in CRC mice (65). The plasma levels of proinflammatory cytokines including CXCL1, Eotaxin, IFN-γ, IL-6, IL-9, IL-12, IL-17A, MCP-1 and TNF-α were increased in *F*. *nucleatum*-treated mice relative to control mice. Accordingly, *F*. *nucleatum* exaggerated inflammation to facilitate HCC development. *F*. *nucleatum* opposed host antitumor immunity by favoring the accumulation of Treg cells and MDSCs and suppressing the infiltration of Th17 cells, NK cells, CD4^+^ T cells and CD8^+^ T cells into liver tissues. *F. nucleatum* treatment deprived the diversity of the gut microbiota, leading to an imbalanced and reorganized intestinal microbial ecosystem. Particularly, *F. nucleatum* administration increased the abundance of *Bacteroides*, *Enterococcus*, *Escherichia*/*Shigella*, *Lactobacillus* and Proteobacteria and reduced the abundance of Bacteroidetes, *Lachnospiraceae_NK4A136_group* and *Ruminiclostridium_9*. Altogether, *F*. *nucleatum* might play a role in supporting CLM by remodeling hepatic immune microenvironment through the modification of the gut microbiota. Disequilibrium of intestinal microbiota controls cancer development by triggering intestinal inflammation, restricting antitumor immunity, or producing tumorigenic metabolites (94, 95). CLM is proposed to be the result from gut microbiota dysbiosis. Future research efforts should be directed to elucidate the inherent mechanisms underpinning the modulation of liver immune response by *F. nucleatum*-induced dysbiosis.

In the gut, the commensal yeast *Candida albicans* could be recognized by the Dectin-3 receptor on the surface of subepithelial macrophage cells (96). Thus, macrophages played an important role in resisting fungal infection and contributed to the maintenance of intestinal homeostasis. Knockout of Dectin-3 caused fungal dysbiosis in colon cancer-bearing mice, as characterized by increased populations of *C. albicans* (66). Dectin-3 deficiency-induced gut dysbiosis could promote colon carcinogenesis. Mechanistically, high load of *C. albicans* promoted hypoxia inducible factor-1 (HIF-1)-dependent glycolysis in macrophages and enhanced IL-7 secretion. IL-7 production from macrophages triggered IL-22 production by ILC3 *via* aryl hydrocarbon receptor and STAT3. ILC3-derived IL-22 elevated the level of p-STAT3 and impelled colon cancer growth in the gut epithelium. Consistently, the IL-22 level in tumor tissues of CRC patients showed a positive association with fungal burden. These findings uncovered a fungal-mediated crosstalk between macrophages and IL-22-generating ILCs during the process of CRC development. The pathways that mediate the effects of commensal fungi on the metabolic program related to inflammatory response in macrophages deserve more attention. It is not clear whether the components or metabolites of commensal fungi can induce energy metabolism changes in macrophages. Currently, the involvement of commensal fungi in cancer development remains largely unexplored, which may stand out as a crucial future research area of investigation.

The importance of the gut microbiota in the regulation of innate immune system during cancer development has been increasingly acknowledged. However, the majority of the insights gained from gnotobiotic animal models. The microbiota-innate immune system interactions in humans are required to be further explored. The range of commensal bacteria that regulate the maturation of innate immune system may be far larger than was previously identified. It is uncertain whether different groups of commensal bacteria adopt common mechanisms to regulate innate immunity. Unlike adaptive immune system, innate immune system is characterized by its expeditious and broad-spectrum response. Innate immunity may act by entirely assessing the activity of the gut microbiota *via* microbial sensing at the tissue-level rather than by responding to particular species of commensal bacteria. Accordingly, it is dispensable to thoroughly reveal how innate immune system recognizes microbial components.

## 4 The gut microbiota and adaptive immunity

### 4.1 Induction of T cell-mediated immunity

Emerging evidence has proven the significant role of the gut microbiota in controlling T cell-mediated immunity (**Table 1**). Introduction of *Helicobacter hepaticus* could suppress CRC growth in mice (67). Mechanistic investigation indicated that *H. hepaticus* activated CD4^+^ T follicular helper (Tfh) cells, increased the number of colon Tfh cells and facilitated the maturation of peritumoral tertiary lymphoid structures. Adoptive transfer of *H. hepaticus*-specific CD4^+^ T cells to Tfh cell-deficient mice could restore antitumor immune responses. Thus, CD4^+^ Tfh cells were required for *H. hepaticus* colonization-induced antitumor immunity. Supplementation of *H. hepaticus* might stand out as a promising therapeutic approach for CRC treatment. Oral administration of *A. muciniphila* prevented colorectal carcinogenesis in mice by inducing antitumor immunity (68). Particularly, *A. muciniphila* increased the proportion of CD8^+^ cytotoxic T lymphocytes (CTLs) in the colon and mesenteric lymph nodes (MLN). It could also activate CTLs in the MLN, which was manifested by TNF-α induction and PD-1 downregulation. *A. muciniphila* may facilitate CTL accumulation by enhancing chemokine secretion by cancer cells. Nevertheless, the underlying mechanisms remain to be revealed. A defined commensal consortium from healthy human feces, consisting of *Alistipes senegalensis*, *Bacteroides dorei*, *B. uniformis* JCM 5828, *Eubacterium limosum*, *Fusobacterium ulcerans*, *Parabacteroides distasonis*, *Parabacteroides gordonii*, *Parabacteroides johnsonii*, *Paraprevotella xylaniphila*, *Phascolarctobacterium faecium*, and *Ruminococcaceae bacterium* cv2, was able to induce the accumulation of IFN-γ-generating CD8 T cells in the intestine (69). The effects of these commensal strains on CD8 T cells were dependent on CD103^+^ DCs and MHC-Ia molecules. Colonization of mice with these commensal strains increased the frequency of IFN-γ^+^ CD8^+^ tumor-infiltrating lymphocytes (TILs) and inhibited tumor growth in a syngeneic mouse model of colon cancer. As expected, commensal bacteria colonization improved the therapeutic efficacy of immune checkpoint inhibitors (anti-PD-1 and anti-CTLA-4) and alleviated their side effects *in vivo*. The 11 commensal strains could be developed into broadly effective biotherapeutics for cancer treatment.

The percentages of Deferribacteres and Proteobacteria were increased and the richness of Actinobacteria, Bacteroidetes, and Tenericutes was reduced in B cell-defective (BCD) mice compared with WT mice (70). Gut microbiota dysbiosis strengthened antitumor immunity in BCD mice with colon adenocarcinoma, as evidenced by expansion of CD8^+^ T cells in tumor tissues. Antibiotic administration attenuated the reinforcement of antitumor activity in BCD mice, suggesting the crucial involvement of the gut microbiota in enhanced antitumor immunity. Intestinal dysbiosis drove the IFN-I signature in mucosal CD8^+^ T cells from BCD mice, leading to increased production of the IFN-I-inducible protein stem cell antigen-1 (Sca-1). The predominant circulation of naïve CD8^+^ T cells between the gut and periphery caused the induction of preactivated Sca-1^+^ naïve CD8^+^ T cells in the periphery and fostered the antitumor CD8^+^ T cell response. Thus, the gut microbiota had the ability to shape peripheral immunity. Naïve T cells show greater potential to replenish the effector T cell pool than fully activated effector T cells (97). Thus, IFN-I-induced naïve CD8^+^ T cells could be preferable for chimeric antigen receptor (CAR) T cell therapy. Considerable work is needed to achieve a clear understanding of intestinal microbiota-mediated regulation of naïve CD8^+^ T cells.

Commensal Clostridiales strains were related to decreased tumor burden in mouse models of CRC (71). Clostridiales strains presented a lower abundance in CRC patients than in healthy controls. An orally applied mixture of four Clostridiales strains (CC4), namely *Anaerostipes caccae*, *Eubacterium hallii*, *F*. *prausnitzii*, and *Roseburia intestinalis*, effectively prevented and treated CRC in mice by promoting the intratumoral infiltration and activation of CD8^+^ T cells and NK cells indicated by increased production of IFN-γ and granzyme B. The anticancer effect of the CC4 mix could be completely abolished in mature B/T cell-deficient mice. The CC4 mix shifted the overall intestinal microbiota composition towards an enrichment in species belonging to the Lachnospiraceae and Ruminococcaceae families. Moreover, microbes belonging to *Anaerostipes* and *Roseburia* genera, as well as *E. hallii*, were increased in CC4-treated mice. Used as a single agent, each of the four strains were effective in diminishing CRC tumor burden. Specifically, *E. hallii* was as effective as CC4, and single supplementation of *A. caccae* or *R. intestinalis* outperformed the CC4 mix. CC4 exhibited higher anticancer efficacy against CRC and melanoma than anti-PD-1 immunotherapy. Since CC4 were efficient in tumors with both low and high quantities of mutations, this bacterial therapy might represent an advantage over immunotherapy. The Clostridiales strains may be exploited as a stand-alone therapy for solid tumors. It is known that the Clostridiales strains could produce butyrate. Additional efforts are warranted to explore whether this anticarcinogenic product acts as an inducer of the tumor inhibition effects of CC4. It is likely that the Clostridiales strains can synthesize certain substances that directly target cancer cells or stimulate antitumor immune cells. Further study is necessary to substantiate this assumption. In summary, this study opens up new possibilities for unearthing effective microbiota-based therapeutics for solid tumors.

Ring-finger protein 5 (RNF5), a membrane-anchored E3 ubiquitin ligase, may play a crucial role in coordinating STING (98). *Rnf5* knockout (*Rnf5*^-/-^) mice had an increased number of *Bacteroides massiliensis* compared with WT mice (72). A lower number of *Lactobacillus* was observed in melanoma-bearing *Rnf5*^-/-^ mice than melanoma-bearing WT mice. The number of DCs was higher in tumors from *Rnf5*^-/-^ mice than WT mice. The expression levels of MHC-II molecules and costimulatory factors (e.g., CD40, CD80 and CD86) were higher in DCs from *Rnf5*^-/-^ mice than in WT mice. ABX could reverse the inhibitory effects of *Rnf5* deletion on melanoma growth, implying that the gut microbiota played an important role in regulating antitumor immunity in *Rnf5*^-/-^ mice. Co-housing of *Rnf5*^-/-^ mice with WT mice resulted in decreased numbers of TILs (e.g., CD4^+^ T cells and CD8^+^ T cells) and DCs, downregulation of MHC-II molecules on DCs, concomitant with reduced frequencies of cytokine-generating T cells. Consequently, the suppressive effect of *Rnf5* depletion on melanoma growth was attenuated after co-housing. Prophylactic transfer of cecal contents from *Rnf5*^-/-^ mice prevented melanoma growth in GF mice partially by increasing the frequency of TILs and improving antitumor cytokine response. Moreover, *Bacteroides rodentium*, one of the bacterial strains enriched in *Rnf5*^-/-^ mice, could elicit antitumor immunity and inhibit melanoma development in GF mice. It was not surprising that *B. rodentium* had a role in immune regulation, since the related family members *B. fragilis and Bacteroides thetaiotaomicron* were capable of stimulating the immune system (99). Collectively, the gut microbiota may contribute to tumor growth inhibition through reinforcement of antitumor immunity in *Rnf5*^-/-^ mice.

The probiotic bacteria, *Escherichia coli* strain Nissle 1917 (EcN), was able to augment the anticancer effect of galunisertib in breast cancer- and HCC-bearing mice (73). Further study indicated that EcN enhanced tumor-specific effector T cell infiltration and induced DC maturation, thus eliciting robust antitumor immune responses. EcN was capable of getting into host intestinal tract where it remodeled the gut microbiota, leading to a shift of intestinal microbes toward specific beneficial bacteria. Specifically, the amount of gut microbiota was elevated in EcN-respond tumor-bearing mice compared to tumor-bearing controls. *Akkermansia*, *Alistipes*, *Bacteroides*, and *Oscillibacter* were significantly increased in EcN-respond mice compared with tumor-bearing controls. *A*. *muciniphila, Alistipes shahii*, *Bacteroides acidifaciens*, *B*. *thetaiotaomicron*, and *Lactobacillus johnsonii* were enriched in EcN-respond mice compared with tumor-bearing controls. Oppositely, the number of *Clostridium_sp.* was decreased in EcN-respond mice. Moreover, *A. muciniphila* showed a positive association with the serum level of IFN-γ while a negative association with the levels of immunosuppressive IL-10 and TGF-β, implying that *A. muciniphila* induced antitumor T cell responses. Furthermore, depletion of the gut microbiota by ABX could abrogate the anticancer activity of EcN in tumor-bearing mice. These observations demonstrated that the gut microbiota might act as a key contributor to EcN-mediated tumor inhibition. *B*. *thetaiotaomicron* was previously found to be a responsible mediator of anti-CTLA-4 treatment *via* activation of the Th1 immune response (99). The enrichment of *B. thetaiotaomicron* might contribute to anticancer efficacy of EcN, and additional work is necessary to corroborate this assumption. The detailed mechanisms underlying the role of specific commensal bacteria in driving antitumor immunity warrant further research. An in-depth investigation of the intricate interactions between the host immune system and the gut microbiota will offer a new possibility to increase the efficacy of conventional anticancer therapies by targeting the gut microbiota.

Intestinal dysbiosis causes the damage of intestinal barriers, allowing microbes to migrate from their original niches to tumor tissues. Zhu et al. (74) revealed that the levels of CD8^+^ T cells positively correlated with overall survival of patients with cutaneous melanoma. Intratumoral bacteria from the genera *Acinetobacter*, *Flammeovirga*, *Gelidibacter*, and *Lachnoclostridium* showed a positive association with infiltrating CD8^+^ T cells. Moreover, these bacteria were positively associated with the levels of CCL5, CXCL9, and CXCL10. Further analysis showed that high load of *Lachnoclostridium* was connected with decreased mortality risk in patients with cutaneous melanoma. These results suggested that the intratumor-residing gut microbiota could influence the clinical outcome in patients with cutaneous melanoma by regulating the levels of infiltrating CD8^+^ T cells and chemokines. Harnessing the intratumor gut microbiota may be a way to treat cutaneous melanoma. It is worth noting that microbial translocation from the gut to tumors needs to be corroborated in preclinical studies. It is still elusive how the gut microbiota modulates the infiltration of CD8^+^ T cells into tumor tissues. Microbial metabolites may also influence TIL activity, which remains to be validated in future studies. Continual efforts are required to evaluate whether *Lachnoclostridium* can improve the efficacy of immunotherapies.

### 4.2 Suppression of T cell-mediated immunity

The gut microbiota may contribute to tumor immune evasion by inhibiting antitumor immunity. Two WT mouse colonies (WT1 and WT2) harbored distinct gut microbial communities (75). WT1 mice had elevated levels of Anaeroplasmataceae, Clostridiales, Erysipelotrichaceae, and Sutterellaceae, while WT2 mice were abundant in Helicobacteraceae and Prevotellaceae. GF mice gavaged with WT1 bacteria developed fewer and smaller tumors than those gavaged with WT2 bacteria. Distinct tumor susceptibilities of WT1 and WT2 mice could be explained by their differences in gut microbiota composition. The enrichment of Prevotellaceae was associated with high tumor burdens, while increased populations of Anaeroplasmataceae and Lachnospiraceae were predictive of low tumor burdens in mouse models of AOM/DSS-induced CRC. WT2 mice possessed increased populations of IFN-γ-producing CD8^+^ T cells relative to WT1 mice before colorectal carcinogenesis, implying that the WT2 microbiota facilitated CRC growth through actions on adaptive immune cells. Knockout of Rag1 abrogated the tumor-promoting effect of gut microbiota dysbiosis in WT2 mice. Depletion of CD8^+^ T cells dramatically prevented CRC development in mice colonized with WT2 microbiota but had no inhibitory effects on tumor growth in mice gavaged with WT1 microbiota. Remarkably, intratumoral T cells in WT2 mice exhibited reduced IFN-γ activity and increased exhaustion. Gut microbiota dysbiosis caused T cell exhaustion by favoring IFN-γ generation by CD8^+^ T cells, which led to destruction of immune surveillance and CRC carcinogenesis. The deficiency of CD8^+^ T cells in mice colonized with WT2 microbiota failed to prevent CRC carcinogenesis, raising the possibility that CD8^+^ T cell-independent mechanisms also mediated CRC tumorigenesis induced by gut microbiota dysbiosis. CD8 T cell response might exert antagonistic effects against tumor development in WT2 mice. The mechanisms linking the causal relationship between gut microbiota dysbiosis and colorectal carcinogenesis warrant in-depth investigation. The involvement of CD8^+^ T cells in inflammation-related carcinogenesis is another important issue that requires special attention. It is obscure how the gut microbiota fosters the activation of IFN-γ-producing CD8^+^ T cells in WT2 mice. Commensal *Bifidobacterium* was able to stimulate DCs to promote CD8^+^ T cell proliferation and IFN-γ synthesis (100). Certain bacterial species may contribute to the expansion and activation of CD8^+^ T cells in colon lamina propria. A possible explanation for the decrease in tumor-infiltrating CD8^+^ T cells is that WT2 microbiome-induced activation of CD8^+^ T cells in intestinal lamina propria causes their exhaustion and impaired antitumor immune system. Therefore, it is essential to identify specific intestinal microbes that have the capacity to prevent T cell exhaustion to restore antitumor immunity. The underlying mechanisms responsible for activation and exhaustion of CD8^+^ T cells necessitate thorough exploration.

ABX could noticeably decrease the quantity and diversity of intestinal bacteria in melanoma- and lung carcinoma-bearing mice (76). Enterobacteriaceae was the most dominant bacterial family in melanoma-bearing mice upon antibiotic treatment, while Burkholderiaceae was enriched in lung carcinoma-bearing mice. Antibiotic-induced dysbiosis could promote the growth of melanoma and lung carcinoma. Importantly, microbiota dysbiosis inhibited tumor endothelial adhesion molecules, including intercellular adhesion molecule-1 (ICAM-1), vascular cell adhesion molecule-1 (VCAM-1), melanoma cell adhesion molecule (MCAM), E-selectin and P-selectin. The counts of effector T cells (e.g., CD4^+^ T cells and CD8^+^ T cells) were also decreased in tumor tissues of dysbiotic mice. The number of TNF-α expressing tumor-infiltrating T cells was markedly reduced in melanoma-bearing mice. The serum concentration of TNF-α was significantly decreased in dysbiotic mice with melanoma, which led to a marked decline in the TNF-α level in tumor tissues. TNF-α acts as a pleiotropic modulator of ICAM-1 (101). Dysbiosis was presumed to decrease the ICAM-1 level by restricting TNF-α secretion, which resulted in the inhibition of T cell trafficking, activation and effector function. Depletion of ICAM-1 could facilitate melanoma growth. Notably, antibiotic-induced dysbiosis could not augment the tumor-promoting potential of ICAM-1 deficiency, suggesting that ICAM-1 mediated dysbiosis-induced tumor development. In addition, TNF-α administration fostered the infiltration of CD8^+^ T cells into tumor tissues and repressed melanoma growth in dysbiotic mice by increasing the expression level of ICAM-1 in the tumor vasculature. These results indicated that commensal microbes in the intestine played an important role in activating antitumor immune response. Antibiotic-induced dysbiosis results in the construction of an immunosuppressive microenvironment, underlining the importance of intestinal microbial communities in the maintenance of host immune homeostasis. The roles of the gut microbiota in modulating antitumor immunity need to be further deciphered. Furthermore, it is worthwhile to determine the commensal microbial species that are involved in dysbiosis-induced tumor progression.

Non-alcoholic fatty liver disease (NAFLD)-related hepatocellular carcinoma (NAFLD-HCC) was characterized by an expansion of Enterobacteriaceae and a decrease in Erysipelotrichaceae and Oscillospiraceae compared with non-NAFLD controls (16). *Bacteroides caecimuris*, *Bacteroides xylanisolvens*, *Clostridium bolteae*, *Ruminococcus gnavus*, and *Veillonella parvula* were enriched in NAFLD-HCC relative to non-NAFLD controls. These five bacterial species had the ability to synthesize SCFAs. As a result, the levels of acetylphosphate and oxaloacetate were markedly higher in feces from NAFLD-HCC patients than in non-NAFLD controls. Butyrate, malonate, and propionate were upregulated in serum samples from NAFLD-HCC patients compared to non-NAFLD controls. Intestinal microbiota from NAFLD-HCC induced the expansion of IL-10^+^ Treg cells and reduced the expansion of cytotoxic CD8^+^ T cells in peripheral blood. Particularly, *B. caecimuris*, *B. xylanisolvens*, and *C. bolteae* were positively associated with effector IL-10^+^ Treg cells, and *B. caecimuris*, *B. xylanisolvens*, *R. gnavus*, and *V. parvula* were negatively linked with CD8^+^ T cells. These bacteria exerted a regulatory effect on adaptive immune responses. Moreover, bacterial extract from NAFLD-HCC inhibited the expansion of monocytes and B cells, alluding to the modulation of antigen presenting milieu. Furthermore, NAFLD-HCC microbiota attenuated the synthesis of proinflammatory cytokines (e.g., IL-2, IL-4, and IL-12) and enhanced the expression of anti-inflammatory cytokine IL-10. NAFLD-HCC microbiota skewed the host immune response towards an immunosuppressive state that promoted cancer development and led to poor clinical outcomes in cancer patients. Butyrate was reported to induce the expansion of Treg cells by upregulating forkhead box p3 (Foxp3) (102). Butyrate also enhanced CD8^+^ T cell effector function (103). The contribution of NAFLD-HCC microbiome-derived butyrate to immune regulation *in vivo* needs systematic research. Manipulation of the gut microbiota and its metabolites may be of benefit in exploring new therapeutic avenues for HCC treatment.

As a key constitute of adaptive immune system, T cells play a vital role in tumor inhibition. Activated T cells can specifically kill cancer cells through their direct cytotoxic effects or production of cytokines to recruit more immune cells (104). T cell-secreted IFN-γ potentiates the antitumor capabilities of other immunocytes (105). IFN-γ also enhances the expression of MHC-I molecules by cancer cells, thus rendering cancer cells ideal targets for T cells (106). Regulation of T cell function is proposed to be an approach to reinvigorate antitumor immune responses. Increasing evidence indicates that the gut microbiota has an intimate relationship with T cell immunity. Given the critical role of the gut microbiota in balancing antitumor versus tumor-promoting immune responses, further studies are required to investigate how commensal bacteria can help tip the balance in favor of antitumor immunity. Since antibiotics influence some critical components of adaptive immunity, ATB should be used with caution in cancer patients that receive immunotherapy. The influence of antibiotics on the immunoregulatory function of the gut microbiota needs to be determined. Until now, there have been limited studies pertaining to the implication and mechanism of actions of each bacterium strain in antitumor immunity. Accordingly, more work is demanded to better understand the interplay between the gut microbiota and adaptive immunity.

## 5 Tumor immunity

On the one side, tumor immunity exerts an important role in preventing cancer pathogenesis (107). The host immune system can specifically recognize and remove cancer cells. Tumor immunity cycle can be divided into the priming and effector phases (108). In the priming phase, cancer-specific antigens are released due to cell death and are seized by dendritic cells (DCs), which causes the maturity and migration of DCs to lymph nodes (109). DCs then present the antigens to MHC-I molecules, which induce T cell activation (110). In the effector phase, the activated T cells then traffic to the tumor site and infiltrate into the tumor tissue, resulting in the elimination of cancer cells (109). T cell-mediated killing of cancer cells in turn impels the discharge of cancer antigens, which drives another tumor immunity cycle (111).

The priming of CD8^+^ CTLs can augment anti-tumor immune responses. CTLs direct cancer cytolysis after recognition of cancer antigens presented by MHC-I molecules expressed by cancer cells (112). CD4^+^ T cells, DCs and NK cells play a critical role in the activation of CD8^+^ T cells (113, 114). Mechanistically, CD4^+^ T cells and NK cells can induce DC maturation and activation through expression of costimulatory molecules and release of cytokines, contributing to the induction of CD8^+^ T cell priming (115). The ligands CD70 and CD80-86 expressed by DCs specifically combine with their corresponding receptors (CD27 and CD28) on CD8^+^ T cells (116). These ligand-receptor interactions are essential for the priming of CD8^+^ T cells. DCs present MHC-I molecules to CD8^+^ T cells to facilitate the formation of effector CTLs (117, 118). CTLs can kill cancer cells *via* Fas ligand (FasL)-mediated apoptosis and perforin/granzyme-mediated granule-exocytosis mechanisms (119). In addition, CTLs exert cytotoxic effects on cancer cells by releasing IFN-γ and TNF-α (120). Activated effector T cell-produced IFN-γ has the ability to activate antitumor M1 macrophages (121). IFN-γ is a key inducer of PD-L1 expression in both M1 macrophages and cancer cells (122, 123). PD-1 receptor is a repressive receptor present on the cell surface of activated T cells (124). The PD-1/PD-L1 axis suppresses the activation of immune cells and represents a momentous mechanism exploited by cancer cells to evade antitumor immunity (125). Treg cell-expressed CTL-associated antigen-4 (CTLA-4) also counteracts the suppressive activity of CD8^+^ T cells, leading to immunosuppression within the TME (113). PD-1/PD-L1 and CTLA-4 are important immune checkpoints that contribute to the exhaustion of effector T cells (126).

On the other side, cancer cells have evolved various strategies to escape immune surveillance (127–129). Immune suppressive cells are contributors to tumor immune evasion. Cancer cell-generated TGF-β promotes the transformation of CD4^+^ T cells into suppressive Treg cells (130). MDSCs, DCs and TAMs may facilitate the initiation and development of cancer by constructing an inflammatory microenvironment (131, 132). MDSCs are proposed to inhibit CTL-mediated antitumor immunity (133). TME-mediated regulation of tumor-infiltrating DCs (TIDCs) impairs antitumor CD8^+^ T cell immunity (134). TAMs favor cancer progression through promotion of angiogenesis and lymphangiogenesis, hypoxia induction and immune suppression (135). Cancer cells can block the detection by the host immune system *via* restricting the expression of MHC-I molecules or targeting the antigen processing and presentation machinery (136). Cancer cells escape from NK cell-mediated cytolysis and induce T cell tolerance by inhibiting the expression of costimulatory molecules (137). On the contrary, cancer cells induce the dysfunction of effector T cells through upregulation of inhibitory molecules (e.g., PD-L1 and CTLA-4) or synthesis of immunosuppressive cytokines (e.g., TGF-β, IL-1, IL-6, and IL-10) (138). Th2-type immunity acts as a promoter of cancer development by counteracting cellular immunity-mediated elimination of cancer cells (139). Particularly, TGF-β and IL-10 drive a shift from the Th1-type response toward the Th2-type response, leading to cancer immune evasion (140). Immunosuppressive enzymes including arginase, indoleamine 2,3-dioxygenase (IDO), and inhibitor of nuclear factor-κB kinase 2 (IKK2) facilitate cancer progression by fostering cancer cell proliferation or driving T cell tolerance (141). Cancer cells trigger the death of cancer-associated lymphocytes *via* caspase-mediated apoptotic cascades (142, 143).

Cancer immune evasion has emerged as a major hurdle for successful anticancer therapies (144–146). Cancer cells exploit multiple immunological processes including enhancement of immunosuppressive cell function, interference with antigen presentation, upregulation of immunosuppressive mediators, induction of immune tolerance and lymphocyte apoptosis. The reciprocal interactions between cancer cells and the host immune system are intricate, but our knowledge regarding this field is still limited. A better understanding of how cancer cells evade immune attack will open up new opportunities for effective therapeutic interventions that can be exploited for the benefit of cancer patients.

## 6 The gut microbiota and cancer therapeutics

Considering the profound effect of the gut microbiota on immune system maturation and antitumor immunity, microbiota supportive therapy shows great promise as a means of augmenting the action of cancer therapies. Moreover, the modifiable property of the gut microbiota makes it possible to develop personalized cancer treatments according to the type and staging of cancer, treatment mode, the microbiota and immune profile in cancer patients.

### 6.1 Cancer immunotherapy

#### 6.1.1 Immune checkpoint inhibitors

**Figure 2:**
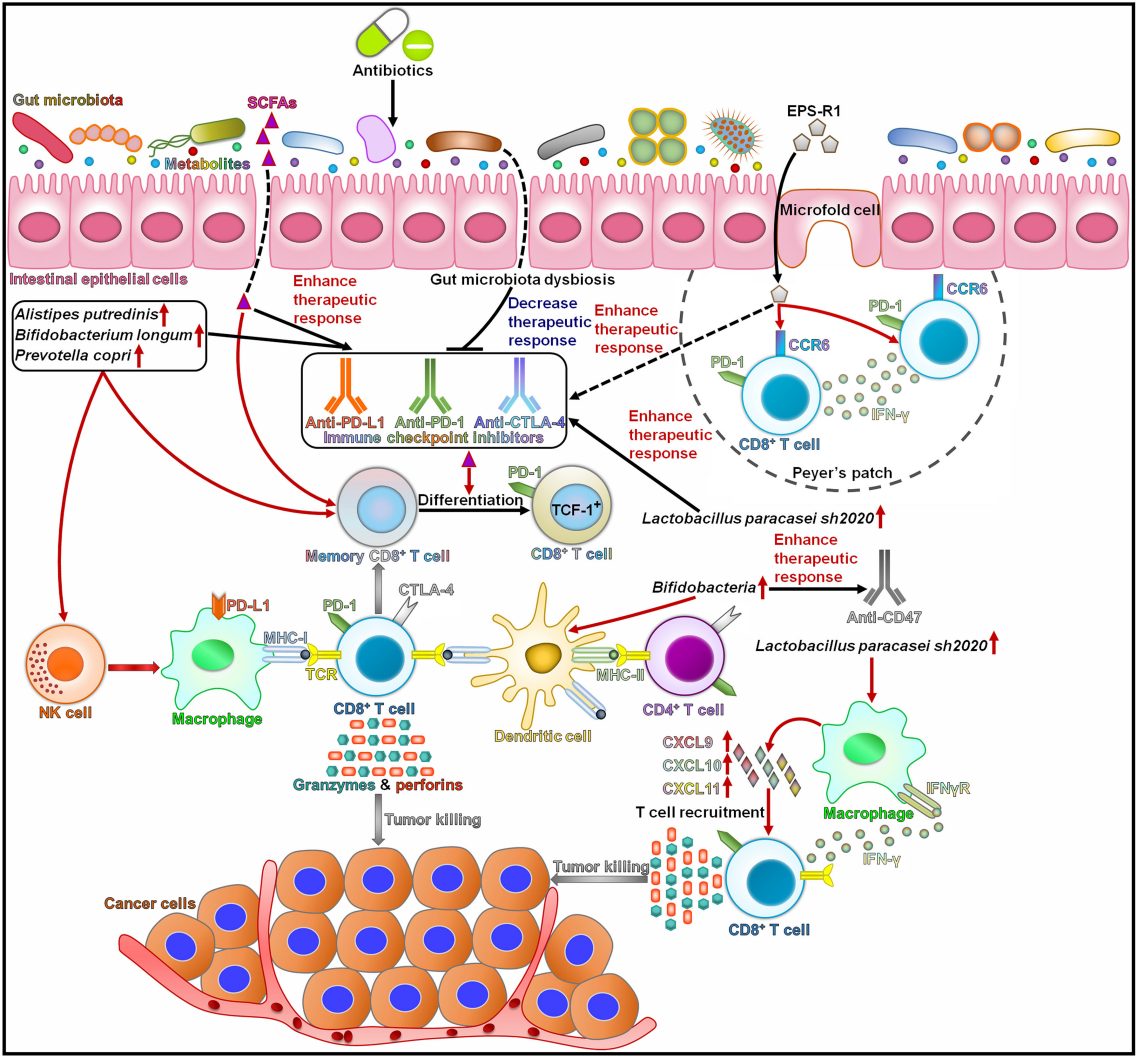
The effect of intestinal microbes and their metabolites on the responses to cancer immunotherapy. Intestinal microbes can affect patients’ responses to immunotherapies through interaction with the host immune system. *Alistipes putredinis*, *Bifidobacterium longum*, and *Prevotella copri* can activate NK cells and memory CD8^+^ T cells and thus enhance the response to anti-PD-1 therapy. SCFAs strengthen the function of memory CD8^+^ T cells and foster their differentiation into TCF-1^+^ PD-1^+^ CD8^+^ T cells, thus improving the efficacy of anti-PD-1 treatment. Gut microbiota dysbiosis induced by antibiotics results in the poor response to immune checkpoint inhibitors. EPS-R1 can augment the anticancer effects of anti-CTLA-4 and anti-PD-1 treatments by inducing CCR6^+^ CD8^+^ T cells in Peyer’s patches and fostering their infiltration into CCL20-expressing tumors. *Lactobacillus paracasei sh2020* promotes the infiltration of IFN-γ^+^ CD8^+^ T cells into tumor tissues by increasing the expression of T cell chemoattractant chemokines. Accordingly, *L. paracasei sh2020* improves the response to anti-PD-1 therapy. *Bifidobacteria* enhances the efficacy of anti-CD47 therapy through activation of dendritic cells. SCFAs, short-chain fatty acids; EPS-R1, exopolysaccharide produced by *Lactobacillus delbrueckii* subsp. *bulgaricus* OLL1073R-1; PD-1, programmed death-1; CCR6, C-C motif chemokine receptor 6; IFN-γ, interferon-γ; TCF-1, T cell factor-1, NK cell, natural killer cell; PD-L1, programmed death-ligand 1; MHC-I, major histocompatibility complex class I; TCR, T cell receptor; CTLA-4, cytotoxic T lymphocyte-associated antigen-4; MHC-II, major histocompatibility complex class II; CXCL9, C-X-C motif chemokine ligand 9; CXCL10, C-X-C motif chemokine ligand 10; CXCL11, C-X-C motif chemokine ligand 11; IFNγR, interferon-γ receptor.

It is generally acknowledged that the composition of the gut microbiota is a key determinant of the response to immunotherapies (147). The use of antibiotics was correlated with worse treatment responses and overall survival in cancer patients treated with immune checkpoint inhibitors (148, 149) (**Figure 2**). Nevertheless, more research is needed to elucidate the exact mechanisms through which ATB-induced gut dysbiosis affects the clinical outcome in cancer patients. Patients with non-small cell lung cancer (NSCLC) who responded to immune checkpoint inhibitors contained higher numbers of *Alistipes putredinis*, *B. longum* and *Prevotella copri* than nonresponding patients (150). By contrast, *Ruminococcus*_unclassified was enriched in nonresponders. The frequencies of memory CD8^+^ T cells and NK cell subsets were increased in the periphery of patients with high microbiota diversity in response to anti-PD-1 therapy. This study provided important implications for assessment of immunotherapeutic response in NSCLC patients. Further studies on the elucidation of the role of the gut microbiota in regulating immunotherapy effect in NSCLC are required. Antibiotic use diminished the efficacy of immunotherapies, including PD-1/PD-L1-based immune checkpoint inhibitors and cytokines, in patients with metastatic renal cell carcinoma (mRCC) (151). By contrast, antibiotic use had limited effects on the clinical outcome of patients treated with mammalian target of rapamycin (mTOR) inhibitors or vascular endothelial growth factor (VEGF)-targeted therapy without prior cytokines. The alternation of intestinal microbial species might affect the effectiveness of immunotherapy in mRCC patients. Several studies also revealed that antibiotic use had a deleterious effect on the clinical outcomes of cancer patients treated with immune checkpoint inhibitors (152–154). The presence of the gut microbiota can affect the therapeutic capacity of immunotherapies, which may be attributable to the immunoregulatory role of the gut microbiota. Nevertheless, it is necessary to illuminate how the gut microbiota synergizes the tumor-inhibiting effects of immunotherapy.

The intratumoral infiltration of C-C motif chemokine receptor 6 (CCR6)^+^ cells was associated with favorable prognosis in some human CCL20-producing cancers (155). Dietary consumption of exopolysaccharide produced by *Lactobacillus delbrueckii* subsp. *bulgaricus* OLL1073R-1 (EPS-R1) induced CCR6^+^ CD8^+^ T cells in Peyer’s patches and fostered the infiltration of these cells into CCL20-expressing tumor tissues in mouse models. The phosphorylated structure in EPS-R1 and a lysophosphatidic acid (LPA) receptor on CD8^+^ T cells mediated the induction of CCR6 expression. The affinity of LPA receptor to EPS-R1 might affect the immunologic effect of LPA. It remains to ascertain whether other receptors are responsible for receiving EPS-R1-derived signals. Moreover, EPS-R1 skewed intratumoral CCR6^+^ CD8^+^ T cells toward an IFN-γ-producing CD8^+^ T cell population that inflamed the tumor tissues. Thus, EPS-R1 reinforced the inhibitory effects of ICB therapies (e.g., anti-CTLA-4 and anti-PD-1 monoclonal antibodies) against CCL20-expressing tumors. On the contrary, CCL20 deficiency counteracted the increased population of CCR6^+^ CD8^+^ T cells and attenuated the anticancer effect of the combined treatment of ICB and EPS-R1 in tumor-bearing mice. Dietary intake of EPS-R1 still intensified the anticancer effect of ICB therapy in GF mice, suggesting that EPS-R1 might act independently of the host gut microbiota. EPS-R1 could be used as an adjuvant therapy in patients with CCL20-producing cancers. The roles of EPS-R1-induced CCR6^+^ CD8^+^ T cells may differ, potentially relying on tumor heterogeneity, the time and duration of ICB therapy, the content and variety of cytokines/chemokines within the tumors and other intratumoral immune cells. Moreover, oral intake of EPS-R1 may regulate the intestine epithelium landscape to augment the immunomodulatory effects of EPS-R1 in Peyer’s patches. Ongoing efforts to determine how EPS-R1 and ICB activate tumor-specific effector CTLs are much needed. The characteristics of IFN-γ-generating CCR6^+^ CD8^+^ T cells induced by EPS-R1 must be completely delineated. In addition, clinical trials should be conducted to assess the therapeutic potential of the ICB and EPS-R1 combination.

A widely consumed dietary fiber, inulin, could increase systemic memory T cell responses and augment the therapeutic efficacy of anti-PD-1 treatment in colon carcinoma-bearing mice (156). Depletion of CD8^+^ T cells caused poor tumor growth control in mice receiving inulin plus anti-PD-1 combination therapy. By contrast, deficiency of CD4^+^ T cells and NK cells had no effects on the anticancer effect of the combined treatment. Thus, CD8^+^ T cells functioned as the key anticancer effector cells. Inulin plus anti-PD-1 treatment increased the numbers of *Akkermansia* and SCFA-producing bacteria *Lactobacillus* and *Roseburia*. Moreover, the abundance of these commensal bacteria and their SCFA metabolites showed an inverse association with tumor sizes. Supplementation of antibiotics led to the worsening of tumor growth control in tumor-bearing mice after inulin plus anti-PD-1 therapy. The gut microbiota might mediate the anticarcinogenic effect of the combined treatment. Moreover, SCFA metabolites from inulin gel enhanced the function of memory CD8^+^ T cells and drove their differentiation into stem-like T cell factor (TCF)-1^+^ PD-1^+^ CD8^+^ T cells, culminating in long-term antitumor immune responses (**Figure 2**). The dietary inulin-based approach represents an attractive and safe strategy to improve the efficacy of immune checkpoint therapies. Microbiota-derived metabolites act as critical messengers between the host immune system and gut microbiota. Additional studies are warranted to explore how inulin gel shapes the host gut microbiota. The mechanisms of action of the gut microbiota and their metabolites in induction of systemic antitumor immunity are worthy of further investigation.

The gut microbiota from healthy individuals conferred enhanced sensitivity to anti-PD-1 treatment in CRC-bearing mice, whereas the gut microbiota from CRC patients tended to diminish the efficacy of anti-PD-1 (157). The percentage of CD4^+^ T cells and CD8^+^ T cells was increased while that of Foxp3^+^ cells was decreased in the tumor tissues of CRC mice colonized with microbiota from healthy individuals. The ratio of Firmicutes to Bacteroidetes was increased in mice colonized with the gut microbiota from healthy controls. The relative abundance of several probiotics, including Bifidobacteriaceae, Erysipelotrichaceae, Lactobacillaceae, and Ruminococcaceae, was increased in CRC mice upon FMT from healthy individuals. These altered microbes might contribute to enhancement of antitumor immunity in CRC. It turned out that *Lactobacillus* cocktail consisting of *L. plantarum*, *L. reuteri*, and *L. paracasei sh2020* improved response to anti-PD-1 therapy in CRC mice. Particularly, *L. paracasei sh2020* showed notable antitumor ability *in vivo*. *L. paracasei sh2020* could promote the expression of T cell chemoattractant chemokines (e.g., CXCL9, CXCL10 and CXCL11), eventually facilitating the infiltration of IFN-γ^+^ CD8^+^ T cells into tumor tissues. Conversely, ablation of CD8^+^ T cells thoroughly offset the effect of *L. paracasei sh2020*. Anti-PD-1 could cause immune-relevant adverse effects in patients with gut microbiota dysbiosis. *L. paracasei sh2020* reversed gut microbiota variations induced by anti-PD-1 treatment, contributing to the restoration of gut homeostasis. *L. paracasei sh2020* combined with anti-PD-1 significantly prevented CRC growth. Promotion of intratumoral T cell infiltration *via* harnessing the gut microbiota has become an area of active research, which is still in the initial stage. *L. paracasei sh2020* could be an effective way to strengthen anti-PD-1 effect in clinical practice. The role of *L. paracasei sh2020* in microbiota regulation needs to be studied in future detail. Continual efforts should be made to better understand the crosstalk between *L. paracasei sh2020* and TME.

#### 6.1.2 CD47-targeted therapy

Colon carcinoma-bearing mice nonresponders to anti-CD47 immunotherapy could recapitulate the response observed in mice responders after cohousing (158). ABX blunted the anticancer activity of anti-CD47 antibody treatment in mice responders. Likewise, GF mice did not respond to anti-CD47 therapy. It was thus supposed that the gut microbiota mediated the anticancer responses of anti-CD47 immunotherapy in colon carcinoma-bearing mice. *Bifidobacteria* specifically targeted tumor tissues and the accumulation of *Bifidobacteria* in the TME improved the capability of tumor inhibition by CD47 blockade in mice nonresponders. Intratumoral administration of ABX counteracted the therapeutic effects of CD47 blockade in mice gavaged with *Bifidobacteria*. Altogether, the anticancer benefit of CD47-based immunotherapy mainly relied on *Bifidobacteria*. Mechanistically, local administration of *Bifidobacteria* triggered the STING signaling and promoted cross-priming of tumor-associated DCs post anti-CD47 therapy (**Figure 2**). Thus, *Bifidobacteria* favored immunotherapy through the STING signaling. Intestinal bacteria can constantly generate metabolites to motivate the STING pathway within DCs. The roles of *Bifidobacteria*-derived metabolites in STING activation merit specific attention. The effect of *Bifidobacteria* and its products on host antitumor immunity also needs to be further dissected. Commensal microbes (e.g., *Bifidobacteria*) inside the TME may synergize with T cell-targeted immunotherapies. Due to its low toxicity and low survival rate in normal tissues, *Bifidobacterium* can be an ideal tumor-targeting bacterium for clinical cancer treatment. Continual studies are indispensable to explore the clinical efficacy of gut microbiota interventions in cancer patients.

### 6.2 Cancer chemotherapy

**Figure 3:**
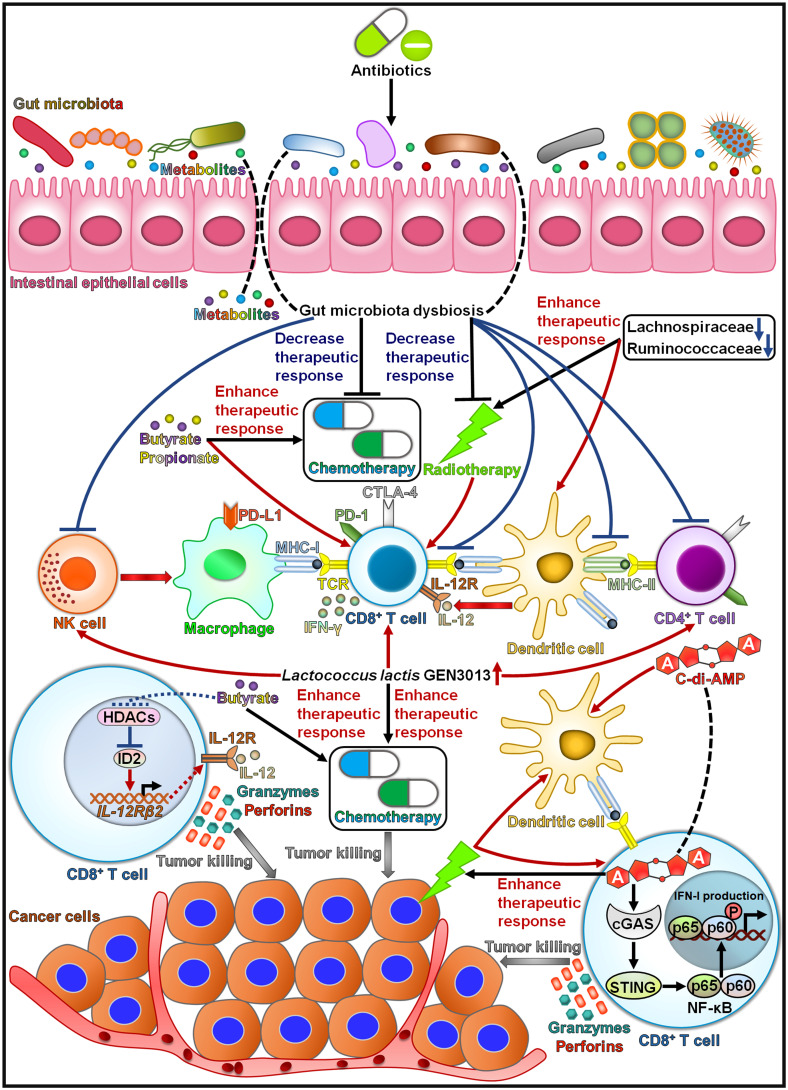
The impact of the gut microbiota on the responses to cancer chemotherapy and radiotherapy. Antibiotic-induced intestinal dysbiosis contributes to decreased therapeutic response to anticancer agents through inhibition of immune cells including NK cells, dendritic cells and CD4^+^ T cells. Depletion of Lachnospiraceae and Ruminococcaceae potentiates the anticancer efficacy of radiotherapy by enhancing antigen presentation and CD8^+^ T cell activity. Gut microbial metabolites butyrate and propionate enhance the anticancer activity of oxaliplatin by inducing CD8^+^ T cell responses. Butyrate can downregulate HDACs to increase ID2 expression, leading to the activation of IL-12 signaling pathway. These events cause the reinforcement of CD8^+^ T cell function and improvement of chemotherapeutic efficacy. *Lactococcus lactis* GEN3013 increases the effectiveness of conventional chemotherapy by promoting the accumulation of NK cells, CD4^+^ T cells and CD8^+^ T cells. Gut microbiota dysbiosis suppresses the activation of T cells induced by radiotherapy, hence restricting the responsiveness to radiotherapy. C-di-AMP synergizes with radiotherapy to facilitate the maturation and presentation functions of DCs, enhancing IFN-β production and CD8^+^ T cell activation *via* the cGAS/STING signaling cascade. NK cell, natural killer cell; PD-L1, programmed death-ligand 1; MHC-I, major histocompatibility complex class I; IFN-γ, interferon-γ; TCR, T cell receptor; PD-1, programmed death-1; CTLA-4, cytotoxic T lymphocyte-associated antigen-4; IL-12, interleukin-12; IL-12R, interleukin-12 receptor; MHC-II, major histocompatibility complex class II; HDACs, histone deacetylases; ID2, Inhibitor of DNA binding 2; IL-12Rβ2, interleukin-12 receptor β2; c-di-AMP, cyclic di-adenosine monophosphate; cGAS, cyclic GMP-AMP synthase; STING, stimulator of interferon genes; NF-κB, nuclear factor-κB.

Antibiotic administration or FMT from antibiotic-treated donors offset the anticarcinogenic potency of trastuzumab in mice with HER2-positive breast cancer (20). Antibiotics (streptomycin and vancomycin) markedly diminished the richness of intestinal bacteria including Actinobacteria, Bacteroidetes, Coriobacteriaceae, Firmicutes, Lachnospiraceae, Prevotellaceae and Turicibacteraceae after trastuzumab treatment. The decreased number of SCFA-producing bacteria (Coriobacteriaceae, Lachnospiraceae, Prevotellaceae and Turicibacteraceae) caused low levels of acetate, butyrate and propionate that facilitated the maintenance of intestinal barrier stability. The decreased production of SCFAs may have an effect on mucosal immunity (159). Vancomycin treatment resulted in a significant increase in the abundance of Proteobacteria and Verrucomicrobia. Depletion of intestinal microbiota inhibited the activation of DCs, CD4^+^ T cells and cytotoxic NK cells in tumor tissues upon trastuzumab treatment *via* an IL12p70-dependent mechanism (**Figure 3**). The abundance of Bacteroidales, Bifidobacteriaceae, Lachnospiraceae, Prevotellaceae, and Turicibacteraceae was higher in HER2-positive breast cancer patients responsive to trastuzumab treatment than nonresponsive patients. The level of *Bacteroidetes* was increased in nonresponsive patients. HER2-positive breast cancer-bearing mice that transplanted fecal microbiota from responsive and nonresponsive patients exhibited similar responses to trastuzumab observed in donor patients. These findings suggested that the gut microbiota exerted a direct effect on chemotherapeutic effectiveness. Specific bacteria altered by antibiotic treatment may be of significance relevance for trastuzumab benefit. Antibiotic-induced alternation of the gut microbiota contributes to the remodeling of tumor immune microenvironment, culminating in the acquisition of trastuzumab resistance and breast cancer development. It is necessary to reveal the mechanisms by which the gut microbiota sustains a favorable immune state for trastuzumab efficacy. The association of the gut microbiota with trastuzumab activity raises the possibility that manipulation of commensal bacteria may represent a potential approach to improve the efficacy of anti-HER2 therapies.

Oxaliplatin retarded tumor growth in colon cancer-bearing mice, whereas antibiotic administration abrogated the efficacy of oxaliplatin (160). Gut microbial metabolites enhanced the anticancer activity of oxaliplatin in antibiotic-treated mice by instigating CD8^+^ T cell responses. Among the microbial metabolites, butyrate and propionate strongly induced the production of IFN-γ by CD8^+^ T cells and strengthened the cytotoxic function of CD8^+^ T cells against cancer cells, suggesting their important contribution to anticarcinogenic effect of the gut microbiota. Inhibitor of DNA binding 2 (ID2) plays a critical role in immune cell differentiation (161). ID2 was significantly upregulated in tumor-infiltrating CD8^+^ T cells upon butyrate treatment compared with naive or activated CD8^+^ T cells. ID2 deficiency abolished the promotion effect of butyrate on CD8^+^ T cell responses, underlining that ID2-dependent pathway was critical for butyrate-induced antitumor immunity. Mechanistic study showed that butyrate increased ID2 expression and promoted CD8^+^ T cell function by suppressing HDACs, and ID2 modulated CD8^+^ T cell function *via* the IL-12 signaling. The *in vivo* study demonstrated that butyrate enhanced the anticancer potentials of chemotherapeutic (e.g., oxaliplatin) and immunotherapeutic agents (e.g., α-PD-L1 antibody) in CAC mice. The role of butyrate in induction of antitumor T cell immunity in cancer patients awaits further substantiation. The effect of other microbial metabolites on chemotherapeutic efficacy should be an important subject of future studies. It is still unclear whether the gut microbiota or microbial metabolites could activate myeloid and CD4^+^ T cells. The underlying mechanisms through which gut microbial metabolites modify the TME necessitate in-depth investigation. *Lactococcus lactis* GEN3013 exhibited anticancer activity against breast cancer, and colon cancer *in vitro* and *in vivo* (162). *L*. *lactis* GEN3013 could improve the effectiveness of conventional chemotherapy (oxaliplatin) as well as immunotherapy (PD-1 blockade). Mechanistically, *L*. *lactis* GEN3013 administration increased the number of immune cells, such as CD4^+^ T cells, CD8^+^ T cells and NK cells in the spleen and TME and decreased the level of suppressor T cells in colon cancer-bearing mice. *L*. *lactis* GEN3013 reprogrammed the host immune system to aggrandize antitumor immunity. Intriguingly, the combination of *L*. *lactis* GEN3013 and bevacizumab or pemetrexed did not exhibit synergistic anticancer effects. It is indispensable to find out whether *L*. *lactis* GEN3013 can improve the efficacy of additional anticancer therapies. The underlying mechanisms that contribute to the promotion effects of *L*. *lactis* GEN3013 on chemotherapy and immunotherapy deserve special attention.

### 6.3 Cancer radiotherapy

Vancomycin treatment caused the elimination of SCFA-producing families, Lachnospiraceae and Ruminococcaceae (163). The altered gut microbiota strengthened the anticancer efficacy of hypofractionated radiotherapy in tumor-bearing mice by promoting antigen presentation and enhancing the functionality of tumor-infiltrating CD8^+^ T cells (**Figure 3**). This effect was abolished by the intake of NaB. Modifying the gut microbiota could be an attractive means to improve radiotherapy-mediated anticancer effects. Gut microbiota dysbiosis was associated with HCC patient response to radiotherapy (164). The diversity of the gut microbiota was remarkably decreased in the non-responder group compared with the responder group. Clostridiales, *Faecalibacterium*, and Ruminococcaceae were dramatically increased in the responder group, while Lactobacillales and *Streptococcus* exhibited greater abundance in the non-responder group. The association between the gut microbiota composition and responsiveness to radiotherapy and the prognosis of HCC patients needs to be verified in further clinical studies with larger cohorts. Antibiotic treatment resisted the antagonistic effects of radiotherapy against HCC cells *in vivo*. Gut microbiota dysbiosis inhibited the accumulation of T cells in the tumor tissues induced by radiotherapy. The levels of infiltrating IFN-γ^+^ CD8^+^ cells were significantly reduced in HCC mice after antibiotic treatment. Transplantation of fecal microbiota could restore the antitumor effects of radiotherapy in antibiotic-treated HCC mice. This rescue effect was abolished in CD8-deficient mice. It was thus inferred that the effects of the gut microbiota on the efficacy of radiotherapy depended on intratumoral CD8^+^ T cells. The level of tumor-infiltrating CD8^+^ T cells was positively associated with the abundance of members belonging to the genus *Faecalibacterium* and negatively associated with that of members from the genus *Streptococcus*. These bacterial taxa might coordinate the activity of tumor-specific T cells in HCC after radiotherapy, and their mechanisms of action merit additional investigation. Mechanistic study showed that gut microbiota dysbiosis impeded antitumor immune responses by blocking antigen presentation and suppressing effector T cell functions *via* the cyclic GMP-AMP (cGAMP) synthase (cGAS)/STING/IFN-I pathway. C-di-AMP, a bacterium-derived STING agonist, was upregulated in the responder group and might act as a critical mediator through which the gut microbiota exerted its immunomodulatory effects. C-di-AMP synergized with radiotherapy to facilitate the maturation and presentation functions of DCs, enhancing IFN-β production and CD8^+^ cytolytic T cell activation in a cGAS/STING-dependent manner. The liver is continually exposed to gut-derived signals *via* the biliary tract, portal vein and systemic circulation. Based on the live-gut axis, the gut microbiota and its metabolites play an important in regulation of radiotherapeutic efficacy in HCC. However, it is still essential to identify the bacterial strains responsible for the high level of c-di-AMP.

### 6.4 The clinical potential of microbiota-based therapy in cancer treatment

The potential benefits of microbiota-based therapy in cancer treatment have been evaluated in several clinical trials. A phase I clinical trial proved the safety and feasibility of FMT in ten patients with anti-PD-1-refractory metastatic melanoma (165). Particularly, FMT treatment could improve clinical responses to anti-PD-1 immunotherapy. Davar et al. (166) reported a phase II clinical trial to assess the therapeutic efficacy of anti-PD-1 responder-derived FMT together with anti-PD-1 in fifteen patients with PD-1-refractory melanoma. This combined treatment was well tolerated and overcame resistance to anti-PD-1 in melanoma patients. In a phase II clinical trial, autologous fecal microbiota transfer (AFMT) could restore the richness and diversity of the gut microbiota in 25 patients with acute myeloid leukemia (AML) treated with intensive chemotherapy and ATB (167). Thus, AFMT treatment was effective in ameliorating gut microbiota dysbiosis in AML patients. A randomized, double-blind, placebo-controlled, phase II study showed that healthy obese donor-derived FMT enhanced chemotherapeutic response and contributed to better prognosis in twelve cachectic patients with metastatic gastroesophageal cancer (168).

Notably, specific transplantation of defined microbial consortia or single microbial species can also augment the efficacy of cancer treatment. A recent randomized phase I trial showed that the *Clostridium butyricum*-including formulation (CBM588) enhanced response to ICB in mRCC patients (169). Progression-free survival was obviously increased in mRCC patients who received CBM588 plus ICB (12.7 months) compared with patients who were treated with ICB alone (2.5 months). Thus, the supplementation of bacterial product may represent an adjunct to anticancer treatment. In a randomized, double-blind, placebo-controlled trial, 99 patients with locally advanced nasopharyngeal carcinoma who underwent concurrent radiochemotherapy (CCRT) were treated with placebo or a probiotic combination that included *B. longum*, *Enterococcus faecium* and *Lactobacillus lactis* (170). The group receiving the probiotic combination (15.63%) had a lower incidence of grade 3 oral mucositis than the placebo group (51.56%). Moreover, the probiotic combination restored the populations of CD3^+^ T cells, CD4^+^ T cells, CD8^+^ T cells, and lymphocytes to normal levels in CCRT-treated patients. These results provided proof-of-concept evidence for the ability of microbiota-centered interventions to reduce treatment-associated side effects and improve the clinical outcome in cancer patients. It is equivocal whether defined commensal consortia could exert the same beneficial effects as FMT treatments. More well-designed interventional clinical studies will be needed to validate these findings. The specific mechanisms of the relationship between the gut microbiota and anticancer treatment remain to be further elucidated. Successive efforts are required to elucidate how the gut microbiota affects physiological and pathological processes in cancer patients. The optimal approaches (e.g., dietary intervention) for modifying or targeting the gut microbiota should be determined. A deeper understanding of the effects of the gut microbiota on drug metabolism must be considered to develop successful microbe-based therapies. Despite many challenges remains, manipulation of the gut microbiota will hopefully become a new avenue for precision cancer treatment.

## 7 Conclusions and future perspectives

The crosstalk between the gut microbiota and tumor immunity has emerged as an important area of research in the field of oncology. The gut microbiota has profound effects on tumor immunity *via* diverse mechanisms. Further studies should focus on elucidating the complex mechanisms that mediate the immunoregulatory function of the gut microbiota. Importantly, concentrated efforts are warranted to systematically clarify the influence of gut microbiota-immune system interactions on cancer development. Preclinical mice models have been widely employed to dissect the colonization of specific intestinal microbes and their interaction with cancer cells and the host immune system, and to provide the proof of principle required to explain and direct clinical studies. Given the significant differences in the gut microbiota between humans and mice, preclinical models can not completely recapitulate the crosstalk between the gut microbiota and the host immune system in cancer patients. Experimental results and conclusions from mouse microbiota studies need to be corroborated by large-scale, multicenter clinical studies.

Manipulation of the gut microbiota by specifically targeting cancer-relevant bacteria, *via* FMT, probiotics, or other approaches, could potentiate antitumor immunity and improve the outcomes of cancer patients. It is worth noting that gut microbiota-targeted therapy still has a long way to go. The comprehensive characterization of the genus or species that can be utilized to enhance protective immune responses is needed. Although some studies have emphasized the potential value of certain microbial species in cancer treatment, the situation in clinical practice can be more intricate. The type and staging of tumor, medications, and a plethora of host factors could disturb the composition of the gut microbiota. Thus, the effect of the gut microbiota on cancer biology and therapeutic efficacy of anticancer treatments may substantially vary across individuals. More clinical studies are demanded to monitor these factors during cancer therapy. An in-depth investigation of the ever-changing gut microbiota in the context of cancer will be helpful in developing personalized treatment strategies for each cancer patient. The gut microbiota has been associated with increased infiltration of immune cells into tumor tissues. However, the TME commonly exhibits an immunosuppressive phenotype, resulting in therapeutic resistance in cancer patients. It is likely that promoting tumor-infiltrating immune cells is not sufficient for instigating effective antitumor immunity. Inducing the transition of these immune cells into anti-tumorigenic phenotypes may be also essential. It remains to verify whether the species considered ineffectual in pre-existing studies can play an adjuvant role when combined with other cancer therapy. Although mounting evidence suggests that the gut microbiota can be adapted to meliorate clinical outcomes in multiple cancer settings, more research efforts need be directed to disclose the mechanisms orchestrated by the gut microbiota to affect patients’ response to cancer treatments. The best-limited consortia makeup that could allow for the optimal therapeutic response in cancer patients must be determined. The feasible strategies to regulate the gut microbiota to improve patients’ response should also be established. Regardless of the curative potential of microbiota modulation for cancer treatment, a more sophisticated understanding of the interaction network between the gut microbiota and tumor immunosurveillance will be pivotal before the gut microbiota can be introduced in clinical cancer therapy.

## Author contributions

MW wrote the manuscript and drew the figures. LZ and WC collected the related papers and revised the manuscript. YZ helped to edit the manuscript. All authors contributed to the article and approved the submitted version.
